# A Prospective Real-World Study Evaluating the Feasibility and Diagnostic Yield of Patient-Recorded Smartwatch EKGs During Palpitations: The WATCHinTIME Study

**DOI:** 10.3390/jcm15083113

**Published:** 2026-04-19

**Authors:** Federico Gibiino, Alberto Boccadoro, Angelo Melpignano, Francesco Vitali, Stefano Clò, Luca Canovi, Marco Micillo, Ludovica Rita Vocale, Elena Marchetti, Michele Malagù, Luca Rossi, Andrea Biagi, Stefano Pieraccini, Paolo Sirugo, Beatrice Dal Passo, Elisa Venturoli, Sara Pazzi, Maria Giulia Bolognesi, Daniela Aschieri, Matteo Tebaldi, Valeria Carinci, Paolo Tolomeo, Gloria Zuccari, Matteo Bertini

**Affiliations:** 1 Cardiovascular Institute, Ospedale degli Infermi, Azienda Unità Sanitaria Locale della Romagna, 48018 Faenza, Italy; federico.gibiino@auslromagna.it (F.G.); alberto.boccadoro@auslromagna.it (A.B.); stefano.pieraccini@auslromagna.it (S.P.); paolo.sirugo@auslromagna.it (P.S.); beatricedalpasso@auslromagna.it (B.D.P.); elisa.venturoli@auslromagna.it (E.V.); sara.pazzi@auslromagna.it (S.P.); 2 Department of Cardiology, University of Ferrara, S. Anna Hospital, Cona, 44124 Ferrara, Italy; angelo.melpignano@edu.unife.it (A.M.); francesco.vitali@unife.it (F.V.); stefano.clo@unife.it (S.C.); luca.canovi@unife.it (L.C.); marco.micillo@unife.it (M.M.); ludovicarita.vocale@edu.unife.it (L.R.V.); michelemalagu@unife.it (M.M.); gloria.zuccari@unife.it (G.Z.); matteo.bertini@unife.it (M.B.); 3 Cardiology Unit, Ospedale Guglielmo da Saliceto Piacenza, 29121 Piacenza, Italy; l.rossi3@ausl.pc.it (L.R.); a.biagi88@gmail.com (A.B.); m.bolognesi@ausl.pc.it (M.G.B.); d.aschieri@ausl.pc.it (D.A.); 4 Cardiology Division, Cardiology Department, Maggiore Hospital, Largo Nigrisoli 2, 40133 Bologna, Italy; valeria.carinci@ausl.bologna.it; 5 Division of Provincial Cardiology, Department of Translational Medicine, University of Ferrara, 44124 Ferrara, Italy; paolo.tolomeo@unife.it

**Keywords:** smartwatch, electrocardiogram, palpitations, arrhythmia, diagnosis, remote monitoring

## Abstract

**Introduction**: Palpitations are one of the most common cardiovascular complaints, affecting approximately 6% to 11% of the general population. Since palpitations often occur sporadically and resolve before medical evaluation, diagnosing the underlying rhythm disturbance requires documentation via an electrocardiogram (ECG) recorded during the symptomatic episode. The standard tool for this purpose has long been the 24-h Holter monitor, which has significant limitations, with diagnostic yields as low as 10% to 15%. **Objective**: This study aims to evaluate the feasibility and diagnostic yield of single-lead ECG recordings from smartwatches in patients presenting with palpitations. **Methods**: From 1 May 2023 to 1 May 2025, we conducted a prospective, real-world cohort study among consecutive adults referred to the University Hospital of Ferrara-based arrhythmia outpatient clinics for evaluation of palpitations. Eligibility required patients to be ≥21 years of age, report palpitations for which ambulatory documentation was clinically indicated, and already own a compatible smartwatch capable of single-lead ECG. Participants were trained to record a 30-s single-lead ECG at the onset of symptoms. Tracings were transmitted securely and independently reviewed by two blinded electrophysiologists. **Results**: Fifty-nine patients were enrolled (mean age 52 years, 64% male). Thirty-one patients (52%) transmitted at least one smartwatch-derived electrocardiographic tracing. Seventy-seven smartwatch tracings were received. Of these, 73 (95%) were interpretable; 57 (78%) showed an arrhythmia, whereas 16 (22%) demonstrated normal sinus rhythm. Four recordings (5%) were non-interpretable. From the 57 arrhythmic tracings, 44 distinct arrhythmic diagnoses were identified. Paroxysmal atrial fibrillation (AF) accounted for 16 episodes. Other diagnosed arrhythmias included atrial flutter (n = 6), paroxysmal supraventricular tachycardia (PSVT) (n = 4), premature atrial complexes (PAC) (n = 6), premature ventricular complexes (PVC) (n = 9), inappropriate sinus tachycardia (n = 12), and second-degree atrioventricular (AV) block type I (n = 4). **Conclusions**: Smartwatch-based ECG monitoring in symptomatic patients is feasible and provides a high diagnostic yield for a broad spectrum of arrhythmias. Unlike large-scale population screening approaches, which generate vast datasets with limited clinical benefit, a symptom-driven strategy applied to carefully selected, educated, and motivated patients proves both clinically valuable and organizationally sustainable. Indeed, the mean number of tracings transmitted per patient was low (1.3), confirming the clinical and operational sustainability of this patient-triggered, real-world approach.

## 1. Introduction

Palpitations are one of the most common cardiovascular complaints, affecting approximately 6% to 11% of the general population. These sensations, often described as fluttering, racing, or pounding of the heart, can be benign but frequently prompt patients to seek medical attention, whether in emergency departments or outpatient clinics. This is due to the potential association of palpitations with underlying cardiac rhythm disturbances that, if undiagnosed or untreated, may lead to severe complications [1]. The causes of palpitations vary widely, ranging from harmless physiological responses to stress, caffeine intake, or exercise, to serious cardiac arrhythmias. Common arrhythmic causes include supraventricular and ventricular premature beats, paroxysmal supraventricular tachycardia (PSVT), atrial fibrillation (AF), atrial flutter, and ventricular tachycardia. Since palpitations often occur sporadically and resolve before medical evaluation, diagnosing the underlying rhythm disturbance requires documentation via an electrocardiogram (ECG) during the episode [2]. The standard tool for this purpose has long been the 24-h Holter monitor, which records heart rhythms continuously over a one-day period. However, this approach has significant limitations, with diagnostic yields as low as 10% to 15%, particularly in patients with infrequent or transient arrhythmias. To overcome these challenges, more advanced monitoring tools have been developed to improve diagnostic accuracy [3,4].

### 1.1. Advances in Prolonged ECG Monitoring

To address the limitations of traditional Holter monitoring, new devices now allow for extended periods of rhythm recording. Long-term Holter monitors and adhesive patch devices can capture continuous ECG data for up to 14 days, substantially increasing diagnostic yields to 66–75% in patients presenting with palpitations. Patient-activated external loop recorders have also proven highly effective, as they allow individuals to document symptomatic episodes directly, enabling a clear correlation between symptoms and ECG findings [5]. Implantable loop recorders (ILRs) represent another significant advancement, offering continuous ECG monitoring for up to three years. These subcutaneous devices are particularly effective for diagnosing bradyarrhythmias and tachyarrhythmias, in patients with unexplained palpitations or recurrent syncope. However, the invasive nature, limited availability, and high costs of ILRs restrict their broader adoption, underscoring the need for more accessible non-invasive alternatives [6].

### 1.2. Atrial Fibrillation and Other Arrhythmias

Among arrhythmias, atrial fibrillation (AF) is the most common sustained rhythm disturbance, affecting millions worldwide. AF is characterized by chaotic and irregular atrial electrical activity, resulting in an irregular ventricular response. Its clinical significance is substantial, given its association with an increased risk of stroke, heart failure, and mortality. Early detection is critical, as timely treatment with anticoagulation can significantly reduce the risk of stroke [7]. AF presents a diagnostic challenge due to its frequent asymptomatic nature, particularly in paroxysmal cases. Other arrhythmias, such as supraventricular tachycardia (SVT), ventricular tachycardia (VT), bradyarrhythmias, and conduction blocks, can also cause palpitations and contribute to diagnostic complexity. Left undiagnosed or untreated, these arrhythmias may lead to circulatory collapse or even sudden cardiac death [7].

### 1.3. Wearable Technology and the Role of Smartwatches in Arrhythmia Detection

Recent advancements in wearable technology have revolutionized cardiovascular monitoring. Smartwatches now provide non-invasive, real-time monitoring of cardiac rhythm during daily activities through photoplethysmography (PPG) and single-lead ECG functionality. PPG sensors detect pulse irregularities using light absorption; algorithms can identify potential arrhythmias such as atrial fibrillation (AF) and alert users [8].

Most smartwatches feature photoplethysmography (PPG) sensors, which measure changes in blood volume using light absorption. PPG algorithms can detect irregular pulse patterns and alert users to potential rhythm disturbances, such as atrial fibrillation. For instance, the Apple Heart Study, involving over 419,000 participants, found that 0.52% of users received irregular pulse notifications, with subsequent patch ECG monitoring confirming AF in 34% of cases [8].

Additionally, many smartwatches now include single-lead ECG functionality. By touching specific electrodes on the device, users can record a 30-s ECG tracing. Studies have demonstrated the high accuracy of these smartwatch-derived ECGs for detecting AF, with sensitivity and specificity exceeding 98% and 99%, respectively, when compared to standard 12-lead ECGs interpreted by cardiologists [9].

### 1.4. Beyond Atrial Fibrillation

While AF has been the primary focus, smartwatch ECGs can detect other arrhythmias such as bradycardia, tachycardia, and complete heart block. For example, smartwatch ECGs have successfully identified bradycardias, tachyarrhythmias, and even ventricular tachycardia. Case reports also document their ability to detect supraventricular tachycardias and advanced conduction abnormalities like complete heart block [10,11,12].

In a study that enrolled 256 patients with a history of cardiac disease presenting to outpatient clinics or emergency departments, a comparison was made between a 12-lead ECG and a single-lead ECG obtained using an Apple Watch provided at enrollment. The single-lead ECG correctly identified all 40 patients with bradycardia, distinguishing between atrioventricular block and sinus bradycardia in 81% of cases. Similarly, all 64 patients with tachyarrhythmias were identified by the single-lead ECG, including two cases of ventricular tachycardia [13].

A 2024 systematic review by Bogár et al., encompassing 57 studies, emphasized the high diagnostic accuracy of smartwatches for life-threatening arrhythmias [14].

Lately Strik et al. enrolled 32 patients with palpitations or presyncope after inconclusive conventional evaluation (normal 12-lead ECG and nondiagnostic 24-h Holter monitoring) [15]. Patients were provided with a single smartwatch model (Withings Move-ECG), and instructed to record and send via email routine ECG weekly and to record an ECG during symptomatic episodes. During a mean follow-up of 17 ± 14 weeks, 640 tracings were recorded: 486 routine (76%) and 154 symptom-triggered (24%). Eighteen patients (58%) recorded ECGs during symptomatic episodes, with the documentations of AF (6 patients), sinus tachycardia (n = 6), normal ECG (n = 2), PVC (n =1), sinus bradycardia (n = 1), and narrow complex tachycardia (n = 1). The study supported the feasibility and potential of smartwatch ECGs in diagnosing paroxysmal arrhythmias (not only atrial fibrillation) during episodes of palpitations and presyncope [15].

In summary these findings, although limited, support the use of smartwatches not only in the diagnosis of atrial fibrillation but also as a tool capable of providing initial documentation of other arrhythmias. 

## 2. Objective

The aim of this study was to evaluate the feasibility and diagnostic yield of patient-recorded single-lead smartwatch ECGs obtained during palpitations. The study had two primary objectives: (1) to quantify feasibility, defined as the proportion of patients able to obtain at least one clinically interpretable smartwatch ECG during symptomatic episodes; and (2) to assess diagnostic yield by characterizing the spectrum of arrhythmias identified following blinded adjudication by cardiologists.

## 3. Materials and Methods

### 3.1. Study Design and Population

This was a prospective, real-world observational cohort study conducted between 1 May 2023 and 1 May 2025 at the arrhythmia outpatient clinics of the University Hospital of Ferrara (Italy). Consecutive adult patients referred for evaluation of palpitations were screened for eligibility.

Inclusion criteria were: age ≥ 21 years, presence of palpitations for which ambulatory rhythm documentation was clinically indicated, ownership of a compatible smartwatch capable of recording a single-lead ECG, and the ability to export and transmit ECG recordings. Details on the smartwatch models supporting single-lead ECG recording at the time of the enrolment are reported in Appendix A.

Exclusion criteria were defined to ensure reliable ECG interpretation and included: presence of implanted cardiac electronic devices (pacemaker, implantable cardioverter-defibrillator, or cardiac resynchronization therapy), baseline ECG abnormalities potentially interfering with single-lead interpretation (e.g., bundle branch block or pre-excitation), known persistent atrial fibrillation, severe tremor or movement disorders impairing signal acquisition, significant cognitive impairment, and pregnancy. At baseline, all participants underwent a standardized cardiological evaluation, including medical history focused on symptom characteristics and triggers, medication review, vital signs assessment, and a resting 12-lead ECG recorded according to institutional standards.

**Figure 1:**
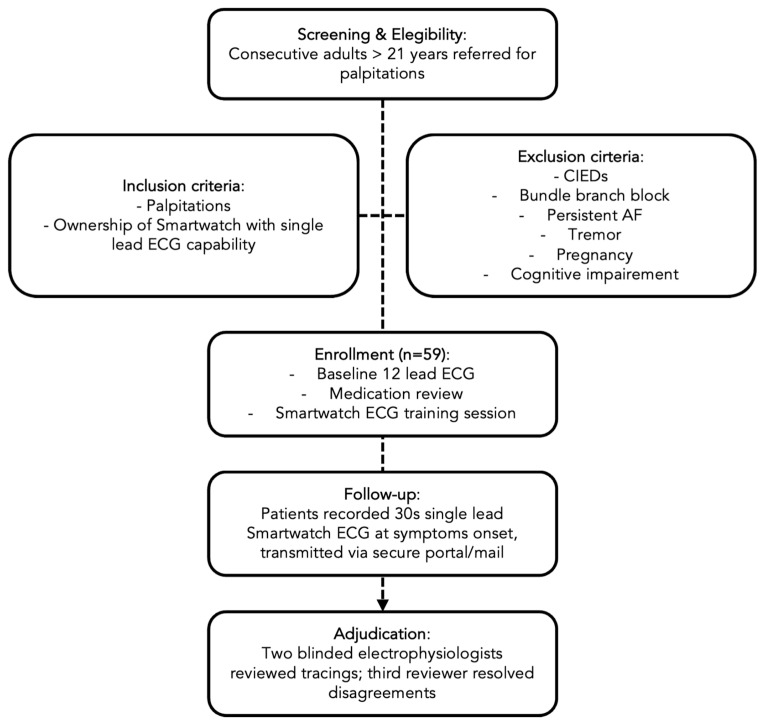
Flow-chart of study methods.

The overall study design is summarized in Figure 1.

### 3.2. Smartwatch ECG Acquisition and Data Transmission

After enrollment, participants received a structured hands-on training session (approximately 15 min) on proper smartwatch ECG acquisition. Patients were instructed to record a 30-s single-lead ECG at the onset of symptoms and, when feasible, after symptom resolution.

To optimize signal quality, participants were advised to perform recordings in a seated position, with the forearm supported on a stable surface and minimal movement, ensuring adequate skin contact with the device electrodes.

At enrollment, all participants recorded a baseline smartwatch ECG under supervised conditions. This baseline tracing was stored and subsequently used as an internal reference to support interpretation of patient-transmitted ECG recordings during follow-up.

Participants were allowed to record and transmit smartwatch ECG tracings at the onset of symptoms for a period of up to 3 months following enrollment.

To minimize motion and myopotential artifacts—well-recognized causes of inconclusive or misclassified tracings in consumer devices—participants were advised to perform recordings in a seated position, with the forearm supported on a stable surface and maintaining adequate skin contact with the device electrodes, according to manufacturer instructions.

ECG recordings were exported as PDF or image files and transmitted via a secure, hospital-approved email system or encrypted upload platform. All tracings were time-stamped and stored in a dedicated study database.

### 3.3. ECG Adjudication

All transmitted ECG tracings were independently reviewed by two experienced electrophysiologists blinded to device type and to each other’s interpretation. Each tracing was first classified as interpretable or non-interpretable based on signal quality.

Interpretable tracings were categorized according to the underlying rhythm as: atrial fibrillation, atrial flutter or organized atrial tachycardia, paroxysmal supraventricular tachycardia, premature atrial complexes, premature ventricular complexes, inappropriate sinus tachycardia, second-degree atrioventricular block type I, or other rhythms.

In case of disagreement, a third senior electrophysiologist adjudicated the final diagnosis.

When tracings were suggestive of atrial flutter or supraventricular tachycardia, patients underwent further clinical evaluation, including electrophysiological study when clinically indicated, in accordance with current guideline recommendations.

### 3.4. Study Endpoints

The primary endpoints were:

- Feasibility, defined as the proportion of patients able to successfully acquire and transmit at least one interpretable smartwatch ECG during symptomatic episodes
- Diagnostic yield, defined as the proportion of interpretable ECG tracings leading to identification of a clinically relevant arrhythmia

Secondary endpoints included:

- Arrhythmia characterization, defined as the distribution and reproducibility of arrhythmic findings across multiple recordings

### 3.5. Ethical Considerations

The study was conducted in accordance with the Declaration of Helsinki and Good Clinical Practice guidelines. The study protocol was approved by the Ethics Committee of Area Vasta Emilia Centro (CE-AVEC), Regione Emilia-Romagna (protocol code: 111/2023/Oss/AOUFe; study code: WATCHinTIME). Written informed consent was obtained from all participants prior to enrollment.

## 4. Statistical Analyses

Descriptive statistics were used to summarize the baseline characteristics of the study population. Continuous variables are presented as mean ± standard deviation, and categorical variables are expressed as frequencies and percentages. Confidence intervals (95%) for proportions were calculated using the Wilson method.

We calculated the number and proportion of patients who successfully transmitted at least one smartwatch ECG tracing and the total number of tracings received. The number and percentage of tracings deemed qualitatively interpretable upon independent review by two blinded cardiologists were determined. Among the interpretable tracings, we calculated the number and percentage that were diagnostic of any cardiac rhythm disorder. For each specific type of arrhythmia identified, the frequency and relative percentage were calculated and reported.

## 5. Results

Descriptive statistics of the 59 patients enrolled are shown in Table 1.

**Figure 2:**
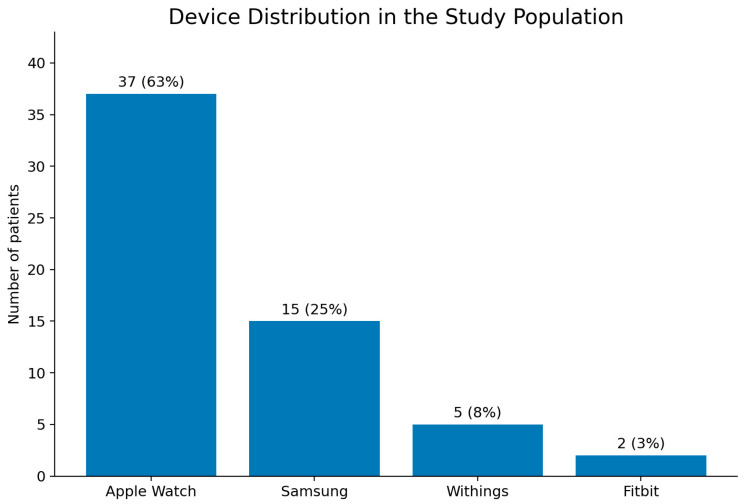
Device distribution in the study population. Bars show the number and percentage of patients using each smartwatch brand.

Between 1 May 2023 and 1 May 2025, we prospectively enrolled 59 patients. The mean age was 52 years (range, 22–75), and 38 participants (64%) were male. The devices used in the study population included the Apple Watch devices (Apple Inc., Cupertino, CA, USA) (62%), Samsung devices (Samsung, Seoul, South Korea) (25%), Withings devices (Withings, Issy-les-Moulineaux, France) (9%), and Fitbit devices (Fitbit Inc., San Francisco, CA, USA) (4%). The devices used in the study population are shown in Figure 2.

We enrolled a highly symptomatic cohort: during the three months preceding enrollment 37% of patients had visited the emergency department for palpitations, and 34% had undergone Holter monitoring. Moreover, the majority had a prior history of arrhythmia: 23 patients (39%) had atrial fibrillation, 5 (8%) atrial flutter, 6 (10%) paroxysmal supraventricular tachycardia (PSVT), 3 (5%) premature ventricular complexes (PVCs), and 2 (3%) premature atrial complexes (PACs). Nineteen patients (32%) were on direct oral anticoagulants (DOACs), 1 (2%) on a vitamin K antagonist, 22 (37%) were receiving beta-blockers, 19 (32%) other antiarrhythmic drugs, and 4 (6%) were treated with levothyroxine.

Fourteen patients (23%) had undergone at least one catheter ablation before enrollment, including 6 for atrial fibrillation, 6 for atrial flutter, 5 for nodal re-entry tachycardia, and 1 for atrioventricular re-entry tachycardia.

During the 3-month observation window, 31 of the 59 enrolled participants (52.5%; 95% CI 40.0–64.7%) successfully transmitted at least one smartwatch-derived ECG tracing to the study core laboratory, providing a total of 77 individual recordings for adjudication (corresponding to an average of 1.3 tracings per enrolled patient).

Among the 77 tracings received, 73 (94.8%; 95% CI 87.4–98.0%) were interpretable.

Of these, 57 (78.1%; 95% CI 67.3–86.0%) showed an arrhythmia, whereas 16 (21.9%) demonstrated normal sinus rhythm.

Four tracings (5%) were non-interpretable due to motion artifacts or incomplete electrode contact.

On a per-patient basis, 30 of 59 patients (51%) had at least one arrhythmic tracing.

Among those who transmitted recordings, the mean number of analyzable episodes per participant was approximately 2.5.

From the 57 arrhythmic tracings, we identified 44 distinct arrhythmic diagnoses.

This difference reflects repeated recordings of the recurrent episodes of the same arrhythmia in individual patients. In other words, repeated tracings from the same individual often reflected the same underlying arrhythmic mechanism rather than distinct arrhythmia types.

Therefore, the analysis distinguishes between the number of diagnostic tracings and the number of arrhythmic events, acknowledging that a single patient may contribute more than one ECG documenting the same arrhythmia (for example, multiple separate episodes of paroxysmal atrial fibrillation).

**Figure 3:**
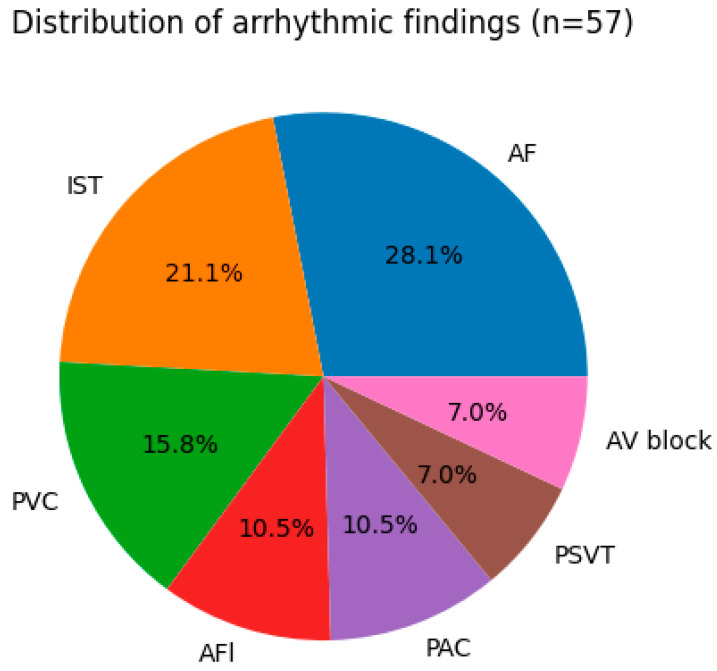
Distribution of arrhythmic findings across smartwatch ECG tracings (n = 57). Percentages are calculated over the total number of arrhythmic tracings (n = 57). Paroxysmal atrial fibrillation (AF) was the most frequent finding (28.1%), followed by inappropriate sinus tachycardia (21.1%), premature ventricular complexes (15.8%), atrial flutter (10.5%), premature atrial complexes (10.5%), paroxysmal supraventricular tachycardia (7%), and second-degree atrioventricular block type I (7%).

Among the 57 arrhythmic tracings, paroxysmal atrial fibrillation (AF) was identified in 16 recordings (28.1%) and was the most frequent arrhythmia. The spectrum of arrhythmic findings included atrial flutter (n = 6; 10.5%), paroxysmal supraventricular tachycardia (n = 4; 7%), premature atrial complexes (n = 6; 10.5%), premature ventricular complexes (n = 9; 15.8%), inappropriate sinus tachycardia (n = 12; 21.1%), and second-degree atrioventricular block type I (n = 4; 7%). The distribution of arrhythmic findings is presented in Figure 3.

Among patients who submitted more than one diagnostic tracing, 82% of subsequent transmissions reproduced the initial arrhythmic mechanism, suggesting stable rhythm phenotypes within individuals over time.

All patients with smartwatch tracings suggestive of atrial flutter or paroxysmal supraventricular tachycardia underwent electrophysiological study with subsequent catheter ablation, and the diagnosis was confirmed in all cases.

**Figure 4:**
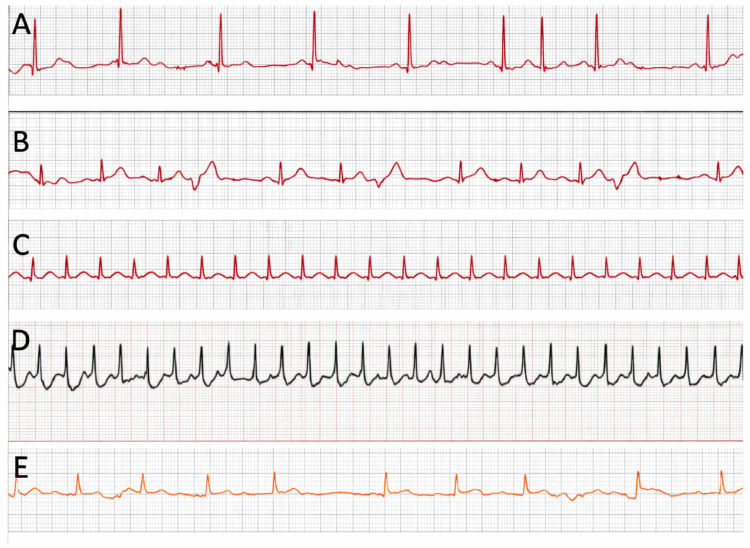
Examples of arrhythmias recorded with the smartwatch during symptoms in the study. (**A**). Supraventricular extrasystole (Apple Watch 5). (**B**). Ventricular extrasystoles with a post-extrasystole compensatory pause (Apple Watch 5). (**C**). Supraventricular tachycardia: atrial flutter was diagnosed after electrophysiological study (Apple Watch 5). (**D**). Supraventricular tachycardia: atrioventricular nodal re-entry tachycardia (AVNRT) was diagnosed after electrophysiological study (Scan Watch 2). (**E**). Second degree type I atrioventricular block (Galaxy Watch 4).

Furthermore, a post hoc review suggested that non-interpretable tracings were most likely related to suboptimal acquisition technique, highlighting the critical impact of proper technique on data quality. Representative examples of smartwatch ECG tracings are shown in Figure 4.

## 6. Discussion

Our cohort consisted of 59 highly symptomatic and relatively young patients (mean age 52 years), 37% of whom had visited the emergency department for palpitations, while 34% had undergone Holter monitoring in the three months preceding enrollment.

Overall, 31 patients (52.5%) transmitted at least one ECG tracing. Among the 77 tracings received, 73 (95%) were interpretable, of which 57 (78%) showed arrhythmias, while four (5%) were non-interpretable. These findings indicate that smartwatch ECG recordings can meaningfully contribute to arrhythmia detection in real-world clinical practice, particularly when recordings are performed during symptoms.

The performance observed in our study was likely supported by the characteristics of our cohort. All participants already owned and were familiar with smartwatch technology, and a high proportion were university (56%) or high-school (39%) graduates. Prior research has demonstrated that wearable device adoption is more common among younger and more educated individuals, favoring correct device use and successful ECG acquisition [16]. This profile probably contributed to both the high interpretability rate and the limited number of unusable tracings.

Our results contrast with those of the prospective study by Strik et al. (Heart Rhythm, 2024), who enrolled 32 patients with unexplained palpitations and provided each with a single smartwatch (Withings Move-ECG) [15]. More than 600 ECGs were collected, but the vast majority were scheduled, asymptomatic “screening” recordings, yielding very few diagnoses [15]. Conversely, our participants were more symptomatic, several had recently required emergency or Holter evaluation, and ECG transmissions were exclusively symptom-triggered. This focused approach resulted in a higher proportion of clinically meaningful diagnoses despite substantially fewer tracings.

The importance of a symptom-driven strategy is further highlighted by the Apple Heart Study, which enrolled over 419,000 predominantly asymptomatic users. Only 0.52% received an irregular pulse notification, and atrial fibrillation was confirmed in just 34% of those undergoing confirmatory patch monitoring [8]. Although the study generated an enormous amount of wearable data, the diagnostic yield was extremely low. These results raise concerns about sustainability for cardiology services tasked with reviewing thousands of tracings with minimal clinical value. In our study, the mean number of tracings per patient was only 1.3, indicating high diagnostic efficiency while maintaining an optimal workload for clinicians. Unlike traditional monitoring strategies, which passively record cardiac rhythm over a predefined time window, smartwatch ECGs allow patients to capture rhythm disturbances at the exact moment of symptom occurrence. This significantly increases the probability of obtaining diagnostically relevant recordings, particularly in the case of transient or paroxysmal arrhythmias. This supports the organizational sustainability of smartwatch-based ECGs when used in well-selected symptomatic patients.

The high reproducibility of arrhythmic findings across multiple recordings further supports the reliability of smartwatch-derived ECGs in characterizing individual arrhythmia patterns over time.

Another notable strength of our investigation is the broad spectrum of arrhythmias detected. Whereas most previous studies have focused almost exclusively on atrial fibrillation, we documented atrial fibrillation (n = 16), atrial flutter (n = 6), paroxysmal supraventricular tachycardia (n = 4), premature atrial and ventricular contractions (n = 6 and n = 9, respectively), inappropriate sinus tachycardia (n = 12), and second-degree type I atrioventricular block (n = 4). This variety underscores the diagnostic value of smartwatches in patients with palpitations and suggests their utility extends beyond atrial fibrillation screening alone.

Smartwatch-derived ECG recordings provided clinically relevant information that supported patient evaluation, although this study was not designed to systematically assess clinical decision-making or outcomes.

Taken together, our findings support a paradigm shift in the role of smartwatches within cardiology. Instead of functioning primarily as population-wide screening tools in largely asymptomatic individuals, smartwatch ECGs may serve as efficient, targeted diagnostic devices when used by symptomatic, technologically familiar patients capable of capturing and transmitting ECGs during episodes. This approach appears both clinically effective and operationally feasible for healthcare systems implementing remote arrhythmia monitoring.

However, smartwatch technology should be viewed as complementary rather than substitutive. Conventional Holter monitoring remains the gold standard for diagnosing infrequent or nocturnal arrhythmias requiring extended continuous recording. Smartwatches excel when rapid, patient-initiated documentation is needed for transient symptoms. Integrating both modalities within clinical workflows may therefore represent the optimal strategy to enhance arrhythmia detection and improve patient care.

## 7. Limitations

This study has several limitations.

First, the sample size is relatively small and reflects a preliminary analysis, which may limit generalizability.

Second, interobserver agreement between the two independent electrophysiologists was not formally quantified, which may limit the reproducibility of ECG interpretation.

Third, smartwatch ECG recordings are inherently limited by their single-lead configuration and may not capture complex arrhythmias requiring multi-lead analysis.

Variability in patient compliance and recording quality may have influenced the proportion of interpretable tracings.

Fourth, a potential selection bias must be acknowledged, as only patients who already owned a compatible smartwatch were enrolled. This cohort is not fully representative of the general population presenting with palpitations, as smartwatch users tend to be younger and more technologically engaged. Moreover, this may reflect underlying differences in education, socioeconomic status, and digital literacy, thereby limiting the generalizability of our findings to broader routine clinical populations.

Lastly, the study employed a single-arm design without a direct, prospective comparison to Holter monitoring. Therefore, we cannot definitively claim the superiority of the smartwatch approach over Holter monitoring. However, our results strongly suggest a potential diagnostic advantage for its use in a selected population. For future research, an ideal design would involve providing the device to a randomized patient cohort and comparing outcomes with a control group using standard diagnostic methods.

## 8. Conclusions

Smartwatch-based ECG monitoring in symptomatic patients who already own and routinely use their device is feasible, effective, and associated with a high diagnostic yield and a very low proportion of uninterpretable tracings. Unlike large-scale population screening approaches, which generate vast amounts of data with limited clinical benefit, a symptom-driven strategy applied to carefully selected, educated, and motivated patients proves both clinically valuable and organizationally sustainable. Indeed, the mean number of tracings transmitted per patient was low (1.3), confirming the clinical and operational sustainability of this patient-triggered, real-world approach.

By documenting not only atrial fibrillation but also a wide range of other arrhythmias, our study suggests that smartwatches can play an important role in the diagnostic workup of palpitations in real-world cardiology practice. This paradigm emphasizes the centrality of symptoms in guiding ECG acquisition and highlights the potential of wearable technology to complement and enhance conventional diagnostic pathways.

Larger studies are warranted to confirm these findings and to further define the role of wearable ECG devices within integrated clinical workflows.

## Figures and Tables

**Table 1:** Baseline characteristics of the study population (n = 59).

Age (mean and range)	52 (22–75)
Male (n, %)	38 (64%)
Hypertension (n, %)	19 (32%)
Type 2 diabetes mellitus (n, %)	2 (3%)
Dyslipidemia (n, %)	14 (24%)
Obesity (n, %)	3 (5%)
Current or former smokers (n, %)	13 (22%)
Prior Emergency Department evaluation for palpitations (n, %)	22 (37%)
Prior Holter monitoring (n, %)	20 (34%)
Prior arrhythmias (history)	
Atrial fibrillation (n, %)	23 (39%)
Atrial flutter (n, %)	6 (10%)
Supraventricular tachycardia (n, %)	6 (10%)
Premature ventricular complex (n, %)	3 (5%)
Premature atrial complex (n, %)	2 (3%)
Drug	
Direct oral anticoagulants (n, %)	19 (32%)
Vitamin K-antagonists (n, %)	1 (2%)
Beta-blockers (n, %)	22 (37%)
Other antiarrhythmic drugs (n, %)	19 (32%)
Levothyroxine (n, %)	4 (6%)
Previous ablation	14 (23%)
Ablation of atrial fibrillation (n, %)	6 (10%)
Ablation of atrial flutter (n, %)	6 (10%)
Ablation of atrioventricular node reentry tachycardia (n, %)	5 (8%)
Ablation of atrioventricular reentry tachycardia (n, %)	1 (2%)
Smartwatch brand used	
Apple Watch	37 (62%)
Samsung	15 (25%)
Withings	5 (9%)
Fitbit	2 (4%)

## Data Availability

The data that support the findings of this study are available from the corresponding author upon reasonable request.

## Abbreviations

The following abbreviations are used in this manuscript:

AF	atrial fibrillation
AFl	atrial flutter
AVB	atrio-ventricular block
ECG	electrocardiogram
ELP	event loop recorder
ILR	Implantable loop recorder
IST	inappropriate sinus tachycardia
PAC	premature atrial complex
PVC	premature ventricular complex
PPG	photoplethysmography
PSVT	paroxysmal supraventricular tachycardia

## Appendix A. List of Devices Allowing Single-Lead ECG Recording with CE Certification at the Time of the Enrollment

- Apple Watch (Apple Inc., Cupertino, CA, USA): Series 4, Series 5, Series 6, Series 7, Series 8 and Ultra. (https://www.apple.com/it/healthcare/apple-watch/, accessed on 1 February 2023).
- Samsung (Samsung, Seoul, South Korea): Galaxy Watch3, Galaxy Watch 4, Galaxy Watch 5 and Galaxy Watch Active2 (https://eu.community.samsung.com/t5/newsroom/fare-un-ecg-con-galaxy-watch/ba-p/3315132, accessed on 1 February 2023).
- Huawei (Huwawei Shenzhen, China): WATCH GT 3 Pro and WATCH (https://consumer.huawei.com/en/press/news/2022/huawei-ecg-app-arrives-in-europe-for-huawei-watch-gt-3-pro-and-huawei-watch-d/, accessed on 1 February 2023).
- Withings (Withings, Issy-les-Moulineaux, France): ScanWatch (https://www.withings.com/be/en/scanwatch, accessed on 1 February 2023).
- Fitbit (Fitbit Inc., San Francisco, CA, USA): Fitbit Sense, Fitbit Sense 2, Fitbit Charge 5 and Google Pixel Watch. (https://help.fitbit.com/manuals/manual_ecg_it.pdf, accessed on 1 February 2023).
